# Acclimatization of symbiotic corals to mesophotic light environments through wavelength transformation by fluorescent protein pigments

**DOI:** 10.1098/rspb.2017.0320

**Published:** 2017-07-05

**Authors:** Edward G. Smith, Cecilia D'Angelo, Yoni Sharon, Dan Tchernov, Joerg Wiedenmann

**Affiliations:** 1 Coral Reef Laboratory, Ocean and Earth Science, University of Southampton, European Way, Southampton SO14 3ZH, UK; 2 Marine Biology Laboratory/Centre for Genomics and Systems Biology, New York University Abu Dhabi, Abu Dhabi, United Arab Emirates; 3 IfLS, Institute for Life Sciences, University of Southampton, Life Sciences Building 85, Highfield, Southampton SO17 1BJ, UK; 4 The Interuniversity Institute for Marine Sciences of Eilat, Eilat, Israel; 5 Department of Marine Biology, University of Haifa, 31905 Mt Carmel, Israel

**Keywords:** chromatic adaptation, light, corals, symbioses, green fluorescent proteins, mesophotic reefs

## Abstract

The depth distribution of reef-building corals exposes their photosynthetic symbionts of the genus *Symbiodinium* to extreme gradients in the intensity and spectral quality of the ambient light environment. Characterizing the mechanisms used by the coral holobiont to respond to the low intensity and reduced spectral composition of the light environment in deeper reefs (greater than 20 m) is fundamental to our understanding of the functioning and structure of reefs across depth gradients. Here, we demonstrate that host pigments, specifically photoconvertible red fluorescent proteins (pcRFPs), can promote coral adaptation/acclimatization to deeper-water light environments by transforming the prevalent blue light into orange-red light, which can penetrate deeper within zooxanthellae-containing tissues; this facilitates a more homogeneous distribution of photons across symbiont communities. The ecological importance of pcRFPs in deeper reefs is supported by the increasing proportion of red fluorescent corals with depth (measured down to 45 m) and increased survival of colour morphs with strong expression of pcRFPs in long-term light manipulation experiments. In addition to screening by host pigments from high light intensities in shallow water, the spectral transformation observed in deeper-water corals highlights the importance of GFP-like protein expression as an ecological mechanism to support the functioning of the coral–*Symbiodinium* association across steep environmental gradients.

## 1. Introduction

Light plays a fundamental role in structuring communities of photosynthetic species [1–4]. In the marine environment, these organisms are subject to gradients in both the intensity and spectral composition of light due to attenuation by the water column [5,6]. As such, communities in deeper water must possess mechanisms to cope with a light environment that is characterized by reduced spectral composition and low intensity. Vertical zonation in marine macroalgae led to the formation of the complementary chromatic adaptation theory, which states that there will be selection for photosynthetic organisms whose pigment absorption most closely matches the spectral composition of the irradiance at that depth [7]. Nevertheless, the complementary chromatic adaptation theory is controversial as other authors have suggested that the observed changes in zonation may instead reflect intensity adaptation (e.g. [8]). Consequently, the implications of the reduced spectral composition on photosynthetic organisms living at depth remain poorly understood despite their profound implications for the structure and functioning of marine benthic communities.

Zooxanthellate corals form one of the most productive marine ecosystems and can be found from shallow waters down to deep mesophotic reefs [9,10]. Their success in oligotrophic tropical waters can be attributed to their symbiosis with dinoflagellates of the genus *Symbiodinium* [11]. The depth structuring of coral communities is primarily driven by light [10] as photosynthesis is a fundamental component of the biology of corals; their symbionts can meet more than 90% of their energetic demands through translocation of photosynthetic products [11]. The depth range of some species can span two orders of magnitude in irradiance [12], indicating that corals and their symbionts must possess the capacity to adapt/acclimatize along a light gradient ranging from the white-light-dominated shallow waters to the blue-range, low-intensity irradiance of mesophotic reefs [6,10].

Coral host pigments from the family of GFP-like proteins have been proposed to play an important role in corals' depth acclimatization and adaptation [13–16]. These pigments alter the coral light field through absorption and re-emission of the incident light or by enhancing coral reflectance and light scattering [15,17]. In shallow-water corals, host-produced green fluorescent protein (GFP) homologues have been experimentally demonstrated to reduce light stress of the algal symbionts in a process known as screening [18]. Screening describes the absorption of light by GFP-like red fluorescent proteins and chromo-proteins in the epidermal tissue that reduces the light intensity reaching the zooxanthellae situated in the gastrodermis [18–20]. However, this does not appear to be a universal function for all GFP-like proteins as strongly fluorescent corals have also been found in deeper waters without high light stress [21,22]. The observations of GFP homologues in low-light environments has led to the hypothesis that these pigment proteins could perform a photoenhancement function by transforming wavelengths outside of the zooxanthellae's photosynthetic action spectrum into wavelengths of peak absorption by the photosynthetic pigments [13,23].

One biochemically distinct class of GFP homologues, in particular, the photoconvertible red fluorescent proteins (pcRFPs), has frequently been observed in low-light and depth-generalist species [21,24–27]. In these species, pcRFPs can contribute up to 7% of the coral's total soluble protein content and are constitutively expressed [21,26,27], in contrast to the light-regulated shallow-water GFPs [28]. pcRFPs are distinct from other GFP classes as they irreversibly switch from a green- to an orange-emitting form in the presence of UV/violet light [25,29,30]. This post-translational modification can result in efficient blue to orange wavelength transformation when a pcRFP tetramer contains both the unconverted (blue absorption, green emission) and converted (green absorption, orange emission) subunits [25,31]. Owing to intra-tetrameric fluorescence resonance energy transfer (FRET) between the subunits, blue excitation of the unconverted form directly stimulates the orange emission of the converted form in a highly efficient manner (up to 100%) [25,31]. The blue-to-orange wavelength transformation does not conform to the existing hypothesis of fluorescent protein functions in deeper waters as it shifts the dominant blue irradiance, most suitable for photosynthesis, to orange light that is weakly absorbed by the dinoflagellate photosynthetic pigments [32]. As such, this wavelength transformation appears counterintuitive for organisms living in a light-limiting environment.

In plants, wavelengths outside of the peak absorption of chlorophyll (the ‘green gap’) are responsible for stimulating photosynthesis deeper in leaf tissues [33]. Blue and red light are readily absorbed by chlorophyll, and therefore penetrate poorly inside leaves, whereas green light excites photosynthesis throughout the leaf due to the weaker pigment absorption and increased scattering [33]. The greater penetration of weakly absorbed wavelengths can enhance productivity relative to the poorly penetrating blue light under equal irradiances [34]. Corals can be considered largely analogous to the leaves of plants as they both contain highly pigmented tissues optimized for photosynthesis. Also, the strong light scattering capacity is comparable between the tissues of plants and corals [35]. As such, the spectral dependency of light penetration could also impact the photobiology of their symbionts. The spectral window between the absorption peaks of the chlorophyll–peridinin complexes in *Symbiodinium* is located at the orange wavelengths [32], which are absent in deeper reefs.

Here, we argue that in corals the intra-tetrameric FRET-mediated, blue-to-orange wavelength transformation by pcRFPs represents an adaptation of the coral host to blue, low-intensity light environments, which benefits the symbionts located deeper in the tissue. This redistribution is critical because common low light acclimatization strategies of corals and their symbionts, namely increased numbers of zooxanthellae and/or higher pigment content [12,27,36], create a packaging effect [37]. The efficient removal of the dominant blue irradiance by the uppermost part of the symbiont community deprives cells in the ‘shaded’ areas below of light. In this study, we experimentally demonstrate that spectral transformation by pcRFPs can exploit the absorption window of zooxanthellae pigments to facilitate enhanced light penetration in the coral tissue to sustain photosynthesis in deeper symbiont layers. Furthermore, we show that red fluorescence is an increasingly important ecological trait in deeper parts of the reef and that the presence of pcRFPs correlates with increased survival under long-term exposure to light fields typical of deep-water reefs.

## 2. Material and methods

### (a) Chlorophyll fluorescence distribution

The vertical distribution of chlorophyll fluorescence was measured to quantify the distribution of light absorption within photosynthetic tissues, as opposed to light availability. The chlorophyll emission recorded corresponds to the fluorescence emission of PSII and, to a lesser extent, PSI. Chlorophyll fluorescence profiles were recorded under different excitation wavelengths in a brown colour morph of *Discosoma* sp. with weak cyan fluorescence by a GFP homologue, and a suspension of zooxanthellae (see the electronic supplementary material, Methods). A brown morph of *Discosoma* sp. was chosen to demonstrate the chlorophyll absorption profile in tissues predominantly pigmented by the zooxanthellae and to avoid spectral cross talk between pcRFP emission and chlorophyll emission. The corallimorpharian was selected due to the ease of dissection, the absence of a skeleton, and a tissue thickness similar to coral species studied. The absence of a calcareous skeleton provides the opportunity to measure the role of pigment absorption on the internal light field without having to account for the reflectance of the coral skeleton. In the Discussion section, we interpret our results from these models in the context of the coral skeleton, and discuss the impact of skeletal reflection on the internal light field and the function of pcRFPs.

Individual *Discosoma* sp. were anaesthetized by the gradual addition of MgCl_2_ over 4 h to a final concentration of 3.75% [27] to avoid contraction and then fixed in 1% paraformaldehyde overnight. The paraformaldehyde solution was prepared with sterile filtered seawater to maintain the pH and osmolarity. Fixation was necessary for chlorophyll fluorescence profiles as tissue movement could alter the optical properties; this ensured consistency between measurements taken at different wavelengths. This particular fixation method was selected as it has previously been used to successfully image fluorescence of coral host pigments and the chlorophyll of their algal symbionts [27].

Vertical profiles of chlorophyll fluorescence in tissues have previously been acquired using cross sections [33]. In the present study, fixed tissue was cut into cross sections, approximately 0.5 cm from the corallimorpharian's mouth, using dissecting scissors. The tissue thickness at the centre of the cross section was 3.5–4 mm. The cross section was subsequently submerged in sterile seawater within a Petri dish. A fibre-optic probe (diameter 4 mm) connected to a Varian Cary Eclipse fluorescence spectrophotometer (Agilent Technologies, Santa Clara, CA, USA) was aligned perpendicular to the surface of the tissue. The tip of the probe was placed 2 mm from the surface of the corallimorpharian, providing illumination (irradiance less than 10 µmol photons m^−2^ s^−1^) in 20 nm wide spectral bands, centred at 400–660 nm. Light of different wavelengths was provided by the built-in xenon flash lamp and monochromators of the Varian Cary Eclipse fluorescence spectrometer. The chlorophyll fluorescence distribution was recorded using an EOS600D camera (Canon, Tokyo, Japan) with a Canon EF 50 mm lens and a macro extension tube (45 mm). The images were taken through a 710 nm (±25 nm) bandpass filter (AHF, Tübingen, Germany). The imaging geometry, spectrophotometer settings and camera settings were maintained across measurements at all wavelengths, with the exception of the camera exposure times. Owing to variation in the intensity of the fluorescence spectrometer's lamp output across wavelengths and to ensure sufficient dynamic range of the red channel while avoiding saturation, exposure times for the images were adjusted according to the intensity of the fluorescence. Integration times for the images ranged from 4 to 12 min (electronic supplementary material, table S1). To avoid any biases due to variation in the excitation intensity and camera exposure times, all chlorophyll emission profiles were characterized relative to the maximum fluorescence intensity within each image, as opposed to the absolute values.

The red channel from each image was post-processed using a background blank image to remove systematic noise, a median filter to remove random noise and normalized to the peak intensity using Matlab (Mathworks, Natick, MA, USA).

### (b) Spectroscopy

*In vivo* fluorescence spectroscopy was performed on the live colonies using the fibre-optic attachment of a fluorescence spectrophotometer [38]. The fibre-optic probe, with a fitted spacer, was placed 0.5 cm from the coenosarc tissue, and the excitation and emission properties of the corals were recorded. The excitation and emission slit widths were 10 nm and 5 nm, respectively.

To obtain the zooxanthellae pigment absorbance spectra, zooxanthellae were isolated from *Oxypora* sp. (red morph), *Euphyllia paradivisa* and *Discosoma* sp. growing in simulated shallow- and deep-water light conditions (see below). Furthermore, zooxanthellae pigment spectra were also collected from red and brown morphs of *Echinophyllia* sp. cultured under shallow-water conditions. Zooxanthellae were extracted as described previously [39], washed and resuspended in sterile filtered seawater. In order to obtain an absorption spectrum representative of the *in vivo* pigments, the extraction was performed in sterile filtered seawater as opposed to solvent extraction, which can cause shifts in the spectra [40], and to avoid scattering effects by the cells. The zooxanthellae were sonicated on ice at 30 s intervals for 5 min. The homogenate was subsequently centrifuged for 40 min at 20 000*g*. Absorbance spectra were recorded using a Varian Cary absorption spectrophotometer. The obtained spectra show strong agreement with symbiont absorbance measurements acquired using an integrating sphere [41].

Measurements of irradiance were collected using a LICOR LI-250A, which measures the photon flux of photosynthetically active radiation (PAR: 400–700 nm) in µmol photons m^−2^ s^−1^.

### (c) Mesocosm experiments

Corals used for this study were maintained in the experimental mesocosm system at the Coral Reef Laboratory at the University of Southampton. Replicate colonies of *Montastrea cavernosa*, *Lobophyllia hemprichii*, *Oxypora* sp. and *Echinophyllia* sp. were cultured in simulated shallow- and deep-water light environments. Under shallow-water conditions, corals were exposed to irradiances of 200 µmol photons m^−2^ s^−1^ provided by a metal halide lamp fitted with a 250 W Aqualine 10 000 burner with a colour temperature of 13 000 Kelvin (Aqua Medic, Bissendorf, Germany). Corals in the deep water simulation received an irradiance of approximately 25 µmol photons m^−2^ s^−1^ of blue light from a 54 W Narva T5 fluorescent lamp (Tunze, Penzberg, Germany; electronic supplementary material, figure S1). The two conditions are herein referred to as the shallow-water and deep-water conditions, respectively. For clarity, discussion of the spectral composition of the 54 W Narva T5 lamp and the 250 W Aqualine 10 000 burner will be referred to as the blue-light and white-light experimental conditions, respectively, with their spectral composition shown in the electronic supplementary material, figure S1. Low-light control conditions were set up under which corals were kept at an irradiance of 25 µmol photons m^−2^ s^−1^ under a distant 70 W Aqualine 10 000 burner (13 000Kelvin, Aqua Medic), creating a light environment that combines the light intensity of the deep-water condition with the spectral composition of the shallow-water condition. Spectral characterization of the pigments within the living coral tissue was conducted using the fibre-optic probe of the Varian fluorescence spectrometer, after 12 months of acclimation.

To assess the effect of spectral quality on the fitness of corals with different tissue concentrations of pcRFPs, colour morphs of *L. hemprichii* and *Echinophyllia* sp. were cultured under the different light conditions (25 µmol photons m^−2^ s^−1^ blue-light, 25 µmol photons m^−2^ s^−1^ white-light control) in flow-through compartments of an aquarium system circulating a waterbody of 1400 l. *Lobophyllia hemprichii* and *Echinophyllia* sp. were selected for the survival experiments as the presence of colour morphs with high and low pcRFP expression in both species enables intraspecific comparison of individuals. The relative pcRFP expression between the different colour morphs was measured by comparing the intensity of pcRFP emission, which strongly correlates with GFP-like protein abundance [28]. For the *L. hemprichii* specimen used in this study, we previously demonstrated that the pcRFPs contributed up to 7.0% to the total soluble protein content of the coral tissue [27]. The red morph of *Echinophyllia* sp. [21] shows red tissue fluorescence with approximately 50% of the intensity of the red *L. hemprichii* specimens (electronic supplementary material, figure S5; figure 3). The survival rates of the brown morphs (pcRFP concentration low) and red morphs (pcRFP concentration high) under the different light environments were recorded for 24 months. The survival rate of individuals was assessed by the presence or the absence of live tissue covering the skeleton.

### (d) Red colour morph distribution

The depth distribution of red fluorescent morphs was assessed using a total of 40 horizontal belt transects performed across six different depths and two reef locations in the Gulf of Eilat (Inter University Institute and Mirador). The optical properties of these waters with depth are well characterized and closely approximate oligotrophic waters for the majority of the year [6,21]. In shallow waters, the spectral irradiance profile is broad and contains wavelengths greater than 600 nm, whereas in the deeper mesophotic reefs, the spectral irradiance spectrum is much narrower and is blue-dominated (centred on 490 nm) [6,21].

The belt transects were 25 m in length and 10 cm in width due to bottom time limitations at the deeper sites. For each transect, only hermatypic corals were surveyed. The transects in the shallow sites (1, 5, 12 and 20 m) were performed at night using a blue torch (NightSea, Lexington, MA, USA) for excitation and yellow filters to mask the excitation light. Owing to the predominance of blue light in the underwater light field at 30 and 45 m, the contrast of the red fluorescence was sufficient for transects to be performed during the day without the need for excitation using the blue torches. The proportion of red fluorescent colonies was recorded and calculated relative to the total number of coral colonies.

## 3. Results

### (a) Chlorophyll fluorescence profiles in *Symbiodinium*-containing tissue and *Symbiodinium* suspensions

**Figure 1.**
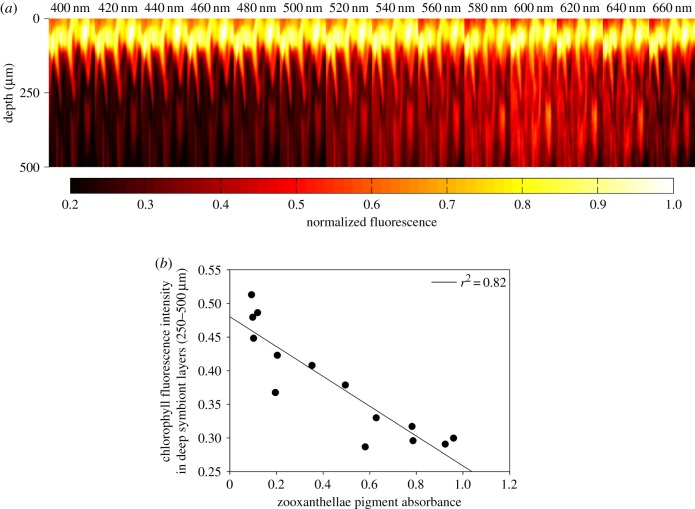
Distribution of chlorophyll fluorescence in response to different excitation wavelengths in symbiont-containing tissue. (*a*) Pseudo-colour images of chlorophyll fluorescence distributions in the gastrodermis of a brown colour morph of *Discosoma* sp. (corallimorpharia) under different wavelengths. The intense fluorescence at tissue depths less than 100 µm correspond to an aggregation of symbionts at the ectoderm/endoderm boundary. Intensities are normalized to a maximum value of 1.0. The width of the imaged area was 1.71 mm. (*b*) Relationship between mean chlorophyll fluorescence intensity between 250 and 500 µm depth in the tissue and the absorbance of symbionts' pigments measured in suspension. The zooxanthellae were extracted from a separate *Discosoma* sp. individual grown under the same conditions as the individual used for chlorophyll fluorescence imaging. The absorbance of the zooxanthellae pigments is normalized to a maximum value of 1.0 and averaged across the 20 nm range of the excitation band. The original maximum absorbance was less than 1.0 and therefore within the linear range of the Beer–Lambert Law.

Using corallimorpharian (*Discosoma* sp.) tissue and zooxanthellae suspensions as models, we assessed light penetration through symbiont communities to explore the potential impact of the deep-water light environment on internal light distribution. Specifically, we assessed light penetration using the distribution of chlorophyll fluorescence as it demonstrates the light absorbed by the photosynthetic pigments and acts as an indicator of the amount of light being used for photochemistry. Our experimental models were exposed to narrow spectral bands (20 nm wide), centred at wavelengths ranging from 400 to 660 nm, and the corresponding distribution of chlorophyll fluorescence was recorded. The chlorophyll fluorescence profile of symbionts *in hospite* shows clear differences in distribution depending on the wavelength of the incident irradiance (figure 1*a*). Under shorter wavelengths of light (400–500 nm) and at 660 nm, the chlorophyll fluorescence in the *Discosoma* tissue is largely concentrated in dense aggregations of zooxanthellae at the oral ectoderm/endoderm boundary with relatively little chlorophyll emission from the symbionts deeper in the tissue cross section. By contrast, excitation with wavelengths between 520 and 620 nm induces a greater proportion of chlorophyll fluorescence from symbionts deeper in the tissue (approx. 125–500 µm) (electronic supplementary material, figure S2), peaking between 580 and 600 nm. The variation in chlorophyll absorption with tissue depth, indicated by fluorescence, correlates strongly with the absorbance spectrum of the photosynthetic pigments of zooxanthellae (*r*^2^ = 0.82; figure 1*b*) and shows a similar pattern of the wavelength distribution with depth as observed in zooxanthellae in suspension (electronic supplementary material, figure S3).

### (b) Optical properties of corals expressing photoconvertible red fluorescent proteins

**Figure 2.**
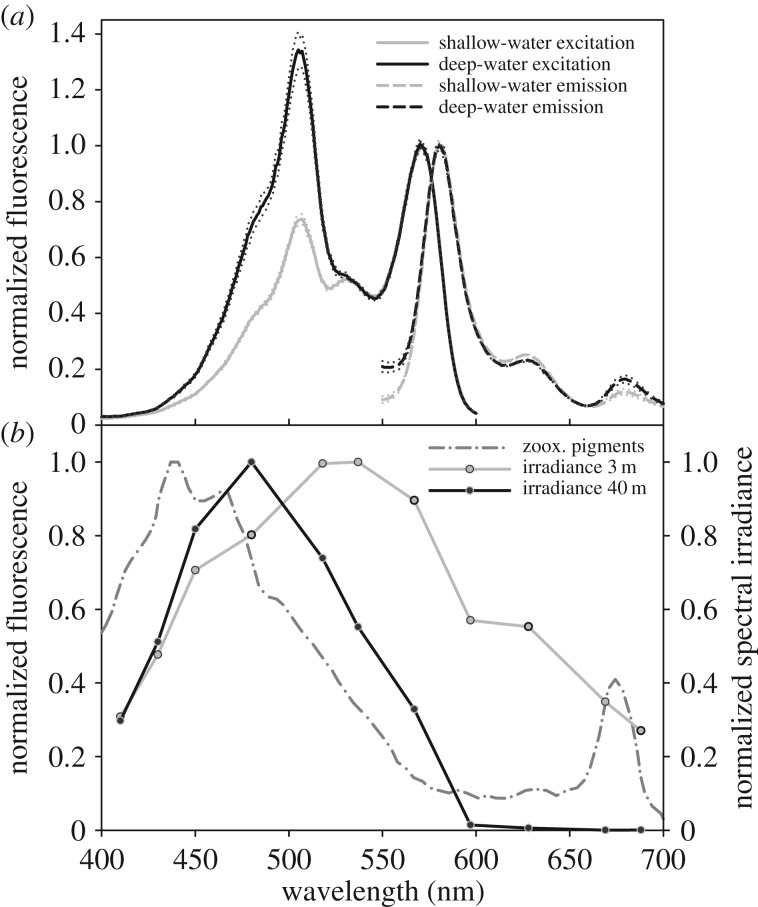
Optical properties of a representative pcRFP in relation to the underwater light field and absorption by the algal symbionts' pigments. (*a*) The *in vivo* excitation (emission at 630 nm) and emission (excitation at 485 nm) spectra of *M. cavernosa* red fluorescence under shallow- and deep-water conditions. The spectra are the mean of five independent measurements and the ±s.d. are shown by the dotted lines. The excitation spectra are normalized to the red excitation peak. The emission spectra are normalized to the peak FP emission at 581 nm. (*b*) The absorbance of freshly prepared zooxanthellae homogenate and representative irradiance at 3 and 40 m depth [42]. The spectra are normalized to a maximum value of 1.0.

We recorded the *in vivo* spectral characteristics of pcRFPs in four species (*M. cavernosa*, *Oxypora* sp., *L. hemprichi* and *Echinophyllia* sp.). The spectra are characterized by emission maxima between 573 and 581 nm and a vibronic sideband at approximately 630 nm (table 1). The excitation spectra are composed of two species, one peaking at approximately 508 nm and the other at 543/570 nm relating to the unconverted and converted forms of the pcRFP, respectively. The relative contribution of each of the two forms is dependent on the incident light field, with the blue-green-absorbing unconverted form contributing a greater proportion under deep-water simulated conditions (25 µmol photons m^−2^ s^−1^ blue light) compared with shallow-water conditions (200 µmol photons m^−2^ s^−1^ white light). A representative example (*M. cavernosa*) is shown in figure 2*a* in the context of the optical properties of shallow- and deep-water light environments and the absorbance characteristics of symbiont pigments (figure 2*b*). The shape of the zooxanthellae's absorption spectra is consistent across host species, colour morphs and light conditions (electronic supplementary material, figure S4). Species-specific variations in the ratio between the green- and red-emitting states of the pcRFPs under the deep-water and shallow-water simulations are outlined in table 1.

**Table 1.** Spectroscopic properties of pcRFPs from four coral species kept under simulated shallow- and deep-water light fields.

coral	green excitation peak (nm)	red excitation peak (nm)	emission peak (nm)	shallow-water conditions green : red excitation ratio	deep-water conditions green : red excitation ratio
*Montastrea cavernosa*	507	571	581	0.7	1.3
*Oxypora* sp.	508	570	581	0.5	0.9
*Echinophyllia* sp.	509	570	581	0.5	0.5
*Lobophyllia hemprichii*	507	543	573	1.7	1.9

### (c) Photoconvertible red fluorescent proteins increase survival in blue-dominated low light fields

**Figure 3.**
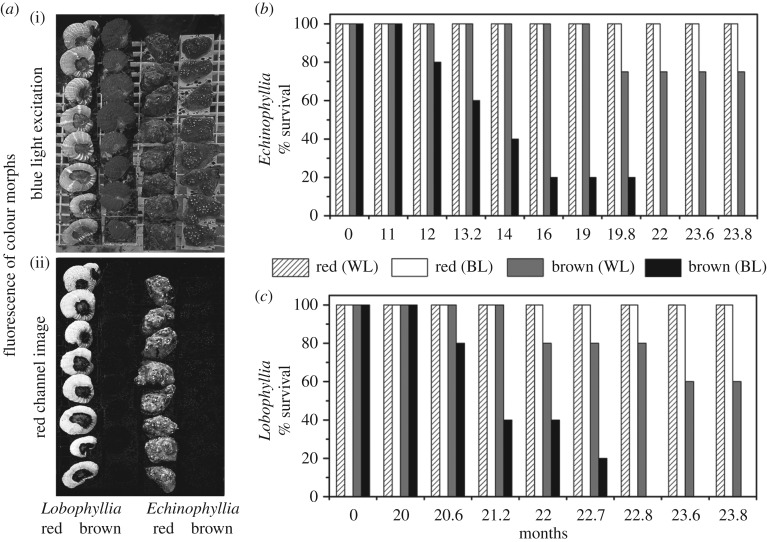
Long-term survival of red and brown colour morphs (red and brown) of *Echinophyllia* sp. and *L. hemprichii* under simulated deep-water light conditions. (*a*) Representative photographic images display the red and brown morphs of the experimental corals under blue-light illumination (i) and their blue-light-excited red fluorescence (ii). (*b*,*c*) Time course of survival of the experimental corals under low-intensity white light (WL) or blue light (BL).

A long-term aquarium experiment was performed to test whether corals benefit from the pcRFP expression in a simulated deep-water light field. Two colour morphs, red and brown (expressing and not expressing pcRFPs, respectively), of *L. hemprichii* and *Echinophyllia* sp. were grown in deep-water (low-intensity blue-light) conditions with a low-intensity white-light environment serving as a control. Fluorescence intensity at the emission maxima for each pcRFP was 2.6 and 19.6 times greater for *L. hemprichii* and *Echinophyllia* sp. red morphs compared with the brown morphs, respectively (electronic supplementary material, figure S5). The red morphs with a high tissue concentration of pcRFPs demonstrated a higher survival rate under low light levels, particularly under the simulated deep water conditions dominated by blue light (figure 3). The brown morphs' survival rate started to decline after 12 and 21 months for *Echinophyllia* sp. and *L. hemprichii*, respectively. At the end of the 2-year experiment, all brown colonies of both species had suffered 100% mortality in the blue-light environment.

### (d) Distribution of photoconvertible red fluorescent protein expressing corals

**Figure 4.**
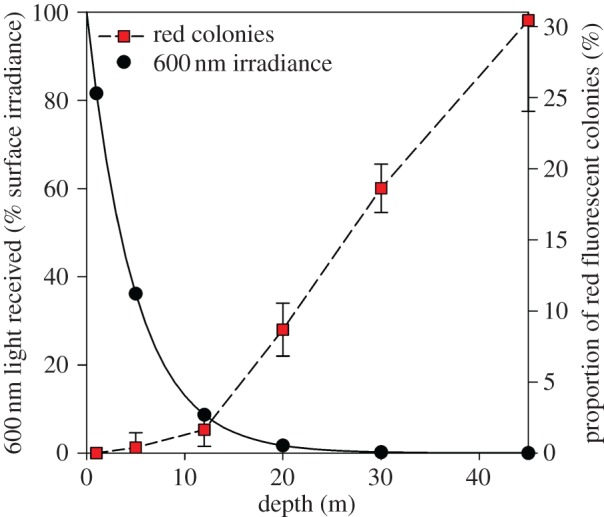
Distribution of red fluorescent coral colonies with depth. Red squares indicate the mean proportion of red fluorescent colonies to the total number of fluorescent colonies at six different depths in the Gulf of Eilat. Error bars show the standard deviation of the means. The mean of colonies per transect is given in the electronic supplementary material, table S2. The approximate proportion of red light (600 nm) received at that depth is indicated by the black circles. The 600 nm light data were interpolated from averaged Kd values for the Gulf of Eilat [43]. (Online version in colour.)

As scleractinian pcRFPs have been observed in species that are typically associated with low-light and deeper-water conditions, we explored the distribution of red fluorescent corals at different depths at two sites in the Gulf of Eilat. Our transect survey revealed that the proportion of red fluorescent colonies within the coral assemblage increases with depth (figure 4). While completely absent in the shallowest surveyed depth of 1 m, the proportion of red fluorescent coral colonies increases with depth, reaching 30% at 45 m depth.

## 4. Discussion

Corals living on deeper reefs inhabit an environment characterized by low light intensities and limited spectral composition of the incident irradiance [6,21]. While responses of zooxanthellate corals to low irradiances are relatively well studied [12,36], the effects of the spectral composition of light on their photobiology are far from being understood. In corals, the typical acclimatory response to low light is an increase in symbiont pigmentation and/or numbers [12,27,36], causing a higher optical thickness of the tissue. Our data suggest the maximized absorption of the blue-green light prevalent at greater depths could come at the cost of poor tissue penetration of these wavelengths. This results in a paradox, where corals' acclimation strategies to maximize absorption decrease the tissue penetration of the blue-dominated irradiance at depth and may consequently limit their capacity to sustain symbiont photosynthesis in deeper tissue areas. The chlorophyll emission cross sections reveal that blue light (less than 500 nm) is indeed strongly absorbed by the photosynthetic pigments, while yellow-orange light (520–620 nm) is used much deeper within the tissue. This is in agreement with internal light field measurements on corals that show orange light dominance in deep tissue [44], and chlorophyll emission profiles of higher plants, where wavelengths between the major absorption peaks of the photosynthetic pigments benefit photosynthesis deeper in the tissue [33]. However, because the orange wavelengths best suited for distributing light evenly across symbiont communities are rapidly attenuated by the water column [21], the conventional low-light acclimation strategy of symbionts and their host may produce detrimental side effects for zooxanthellae in deeper tissue of corals in low-intensity, blue-light environments.

pcRFPs are well suited to overcome the challenges imposed by the blue-dominated deep-reef light fields. These protein pigments are located in the epidermis [27] and therefore can transform the spectral composition of the incident light prior to absorption by the coral's symbionts. In all species studied here, the unconverted green-emitting form of the pcRFPs absorbs blue-green light and can subsequently transform it into orange-red wavelengths through intra-tetrameric FRET to the photoconverted red-emitting form of the protein [31]. Thus, these protein pigments provide a mechanism to overcome the challenges imposed by the deep reef light field by transforming the abundant blue-green wavelengths that spread poorly in coral tissue into wavelengths that provide greater tissue penetration before absorption for photosynthesis. The wavelength transformation mechanism by pcRFPs is in stark contrast to previously hypothesized photoenhancement functions for fluorescent pigments in low-light corals as it initially depletes, as opposed to enriches [13,23], the coral's internal light field in wavelengths optimally absorbed by the photosynthetic pigments in favour of greater light penetration. However, the red-shifted wavelengths can still be used by coral symbionts because the probability of them being absorbed is promoted by multiple scattering within the coral tissue and reflection by the skeleton [37,45–48].

The use of corallimorpharian tissue and zooxanthellae suspensions as models to assess light penetration does not account for scattering by the coral skeleton; however, this diffuse scattering would act to further homogenize the light distribution of those wavelengths that penetrate deeply. Wangpraseurt *et al*. [44] have shown that the PAR availability decreases with tissue depth down to the skeleton and that the available light at the tissue-skeleton interface is dominated by orange-red light (550–650 nm). Despite the absence of a skeleton in corallimorpharian tissue, the chlorophyll fluorescence profiles observed are consistent with scalar irradiance measurements made in corals [44,49] and demonstrate that the wavelengths previously observed to penetrate deeply are also absorbed by the symbionts' photosynthetic pigments.

Exposing replicate corals to different light environments demonstrated how the excitation properties of the pcRFPs are ‘tuned’ to the light field. Reduced photoconversion in the deep-reef light conditions increased the contribution of excitation through the blue-absorbing unconverted green chromophore to the orange emission via FRET-mediated wavelength transformation. The efficiency of blue-to-orange wavelength transformation will increase with depth as photoconversion is dependent on the exposure to near UV/violet light in the environment [21,26,31], which decreases in a depth-dependent manner [21]. The photoconversion efficiency of a pcRFP is dependent on the specific pcRFP's biochemistry and protein environment [50]. These differences in photoconversion efficiency among pcRFPs could help different species to exploit distinct light niches found along depth gradients. The dependence on post-translational modification of the constitutively expressed pcRFPs [21,26] is unique to this family of coral GFP homologues and is distinct from photoprotective shallow-water GFP-like proteins that are regulated at the transcriptional level [28].

The ecological importance of pcRFPs is established here through long-term manipulation experiments and field surveys. In our experiments, red colour morphs (high pcRFP expression) outperformed other colour morphs in the blue-light environments characteristic of mesophotic reefs. Our experimental data are consistent with field surveys where the increasing proportion of red morphs with depth correlates with the loss of red wavelengths from the underwater irradiance. These observations highlight the fundamental importance of the spectral composition of light environments for photosynthetic organisms and their community structure in the marine realm. The experimental observations suggest that shallow-water species without specific chromatic adaptations may not be able to use mesophotic reefs, which could act as refugia for corals threatened by climate change in their shallow-water habitats.

It is important to note that the competitive advantage of pcRFP expression is subtle, as indicated by the long response times in our laboratory experiment and the coexistence of red and non-red fluorescent corals at different depths. The subtlety is likely to reflect the trade-offs associated with pcRFP expression, such as loss of photons related to their quantum yield [27] and the energetic costs of maintaining high pigment concentrations in the tissue. While the production of these pigments is comparatively cheap due to the low turnover rates and the autocatalytic formation of the functional pigment [26], the metabolic cost is likely to be high because multi-copy gene arrays are required to sustain high tissue concentrations [20]. It is well established that scattering by pigment granules, coral tissue and the skeleton can strongly influence the internal light environment [15,17,35,45,48]. Subsurface maxima in scalar irradiance [44,49] resulting from multiple scattering could enhance pcRFP excitation, therefore improving wavelength transformation efficiency. This improvement in efficiency could potentially reduce requirements for high pcRFP expression and lower the metabolic demands for maintaining the pigment array. As established for the photoprotective pigments in shallow-water corals, colour polymorphism can also help to balance the ecological benefits and metabolic constraints of the costly pigment production [20]. In the case of pcRFP-containing corals, the highly pigmented individuals of the population could gain advantages with increasing depths. In contrast, the less pigmented variants may benefit in shallower water because they can invest more energy in other processes that may result in faster growth or higher reproductive output. By this mechanism, the coral–symbiont associations can extend their distribution range along the steep light gradients of coral reefs and inhabit more ecological niches.

Our results underline the functional importance of the underwater light environment's spectral composition and its impact on the ecology of marine photosynthetic communities. In corals, the reduction in spectral composition has led to the development of chromatic adaptations that optimize the internal light field for the benefit of their symbiotic partners. While the impacts of light quality in terrestrial environments are well studied, the complex interactions between internal and external light gradients in submerged environments are greatly underappreciated, and further study could help to overcome controversial discussions about the ecology of marine photosynthetic organisms.

## Supplementary Material

Supplementary Information

## Data accessibility

The data used to create the figures in this manuscript are deposited in the Dryad repository (http://dx.doi.org/10.5061/dryad.19sq4) [51].
