# Supplementary material for: Acclimatization of symbiotic corals to mesophotic light environments through wavelength transformation by fluorescent protein pigments
Source: Proc Biol Sci. 2017 Jul 5;284(1858):20170320. doi: 10.1098/rspb.2017.0320 (PMC5524488; doi:10.1098/rspb.2017.0320)
Supplement: Supplementary Information [file rspb20170320supp1.pdf]

Acclimatisation of symbiotic corals to mesophotic light environments through wavelength transformation by fluorescent protein pigments

Edward G Smith[1,2], Cecilia D'Angelo[1,3], Yoni Sharon[4], Dan Tchernov[4,5], Joerg Wiedenmann[1,3]

[1] Coral Reef Laboratory, Ocean & Earth Science, University of Southampton, European Way, SO143ZH, Southampton, UK

[2] Marine Biology Laboratory / Centre for Genomics and Systems Biology, New York University Abu Dhabi, Abu Dhabi, UAE

[3] IfLS, Institute for Life Sciences. University of Southampton, Life Sciences Building 85, Highfield, SO171BJ, Southampton, UK

[4] The Interuniversity Institute for Marine Sciences of Eilat, Eilat, Israel,

[5] Department of Marine Biology, University of Haifa, 31905 Mt. Carmel, Israel

DOI: 10.1098/rspb.2017-0320

Supplementary Information

Supplementary Methods

Chlorophyll fluorescence distribution in a zooxanthellae suspension

Zooxanthellae were isolated from *Euphyllia paradivisia* grown under the deep water conditions and employed as a model for the characterisation of coral symbiont absorption. These symbionts have been characterised as ITS2 type C1 [1], the same ITS2 types as the *Discosoma sp.* used in this study and have comparable absorption properties. Tentacles were cut from two polyps and the tissue was disrupted using a potter homogeniser. The tissue slurry was filtered using a 0.1mm mesh and the homogenate was then centrifuged at 700xg for 5 minutes to pellet the symbionts. The symbiont pellet was resuspended in 50ml of sterile

filtered seawater and centrifuged again. This wash step was repeated twice. After the wash steps, the zooxanthellae pellet was resuspended in 1ml sterile filtered seawater and the concentration of zooxanthellae was determined using a haemocytometer and epifluorescent microscope. The concentration of the zooxanthellae suspension was then adjusted to $5x10^6$ cells cm^{-3}. This volume was chosen as it corresponds to the aerial density of symbionts recorded in *Oxypora sp.* ($5x10^6$ cells cm^{-3}) but extended over a larger distance (10mm) compared to the tissue thickness (~1mm, contracted) to improve visualisation.

The zooxanthellae suspension was loaded into a cuvette and the zooxanthellae were kept in suspension by gentle mixing with a magnetic bead on a stir plate. The fibre optic probe of a Cary Eclipse fluorescence spectrophotometer (Agilent Technologies, CA) was inserted into the suspension and the Xenon lamp and the optics of the instrument were used to provide 20nm wide wavebands to excite the zooxanthellae in suspension at central wavelengths ranging from 400 to 660nm (Supp. Fig. 6). Images of chlorophyll fluorescence were taken with a Canon EOS600D through a 710nm (±25nm) filter.

Supplementary Figures

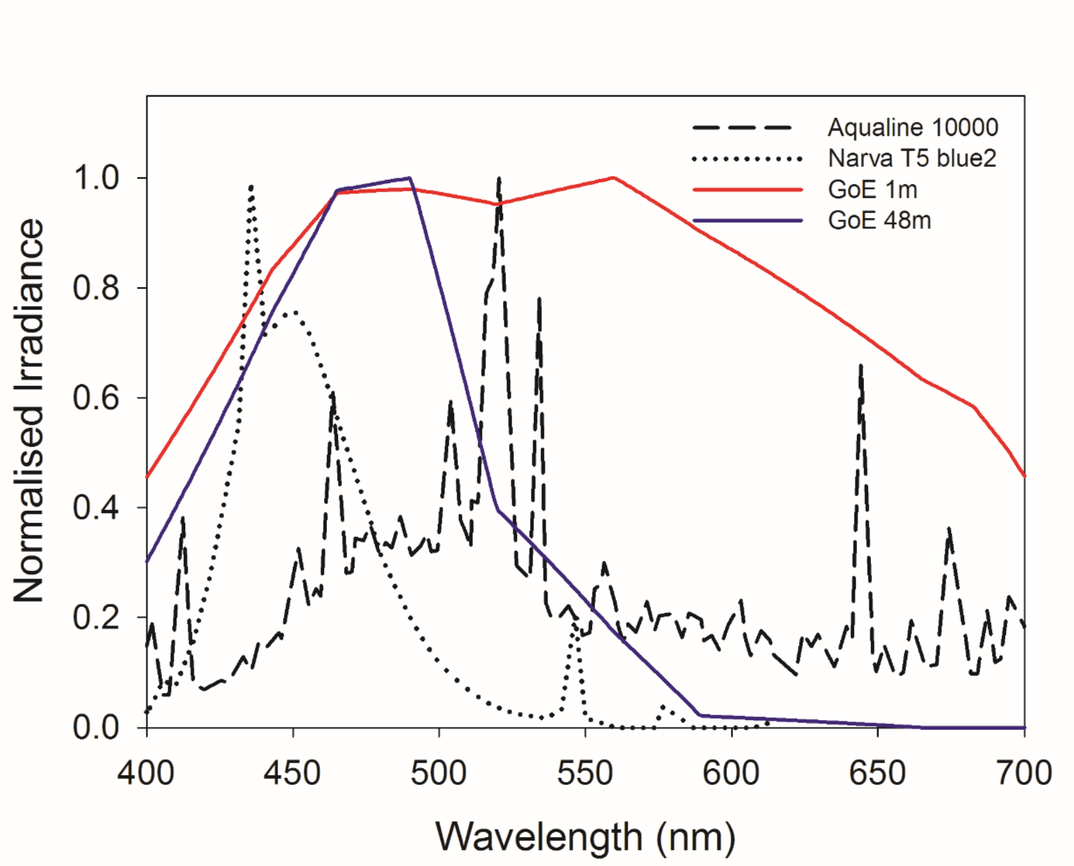

Supplementary Figure 1. Normalised spectral output of the light sources used in this study and representative spectra from the Gulf of Eilat (GoE; [2]). The Aqualine 10000 (13000K) was used for the shallow water light conditions whereas the Narva T5 blue2 was used for the deep water conditions.

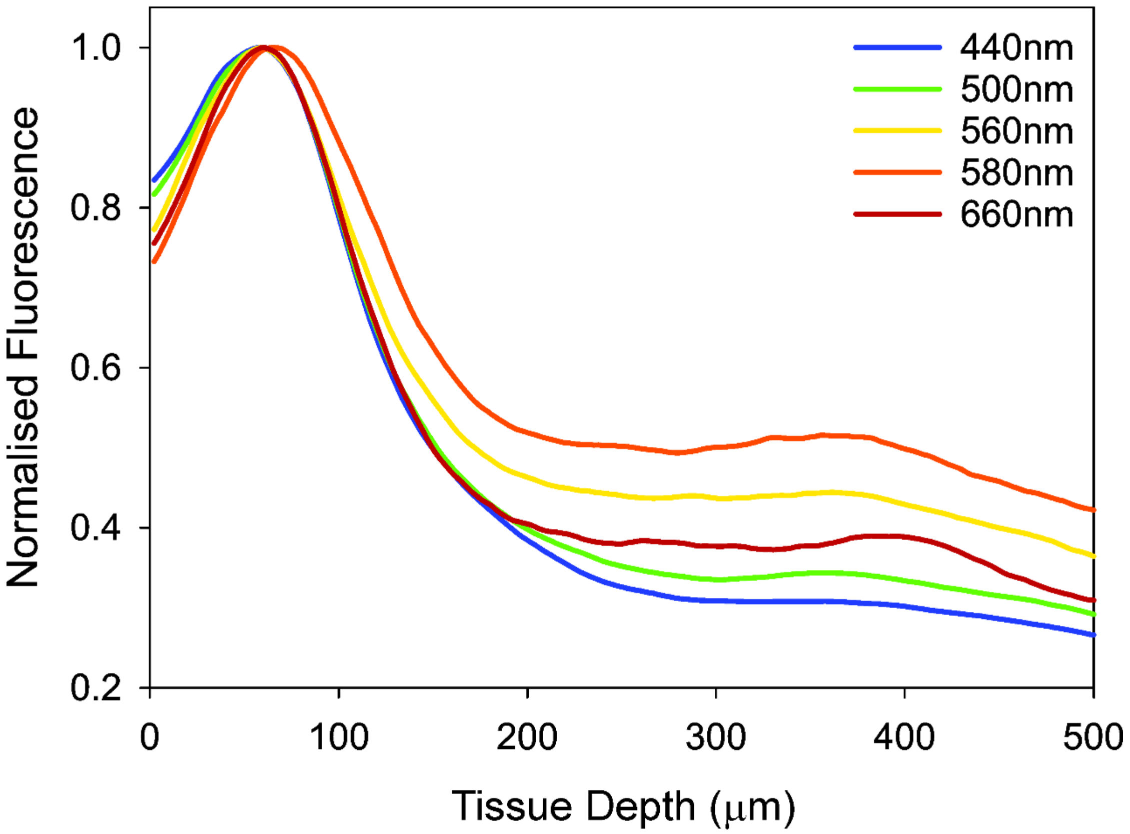

Supplementary Figure 2. Mean chlorophyll fluorescence distribution at different depths throughout the corallimorpharian tissue at different excitation wavelengths. Data are normalised to a maxima of 1.0.

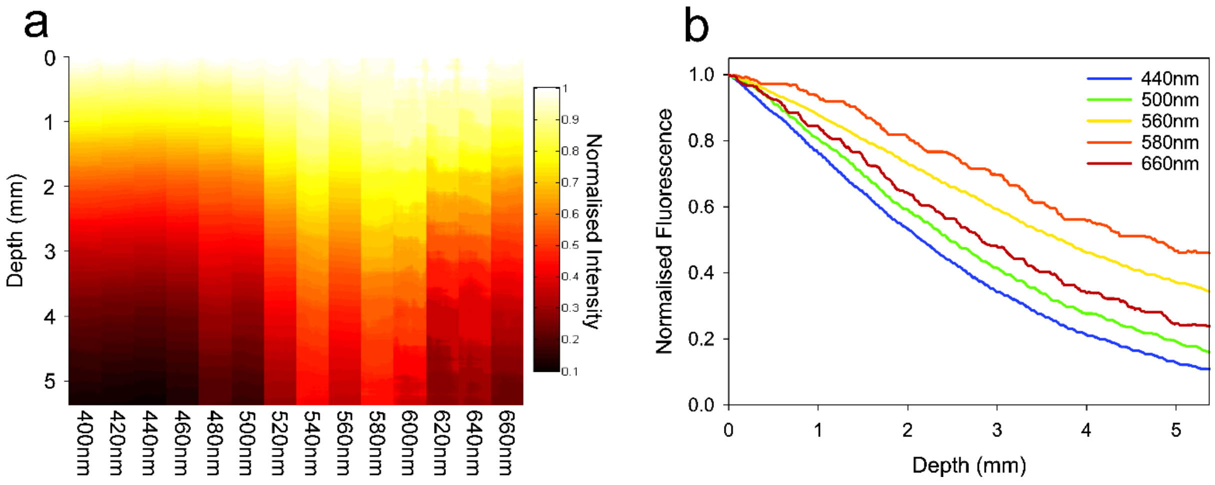

Supplementary Figure 3. Chlorophyll fluorescence through a zooxanthellae suspension. (a) Images of chlorophyll fluorescence distribution under different excitation wavelengths. Intensities are normalised to a maximum value of 1.0. (b) The corresponding mean chlorophyll fluorescence at different depths within the suspension under different excitation wavelengths.

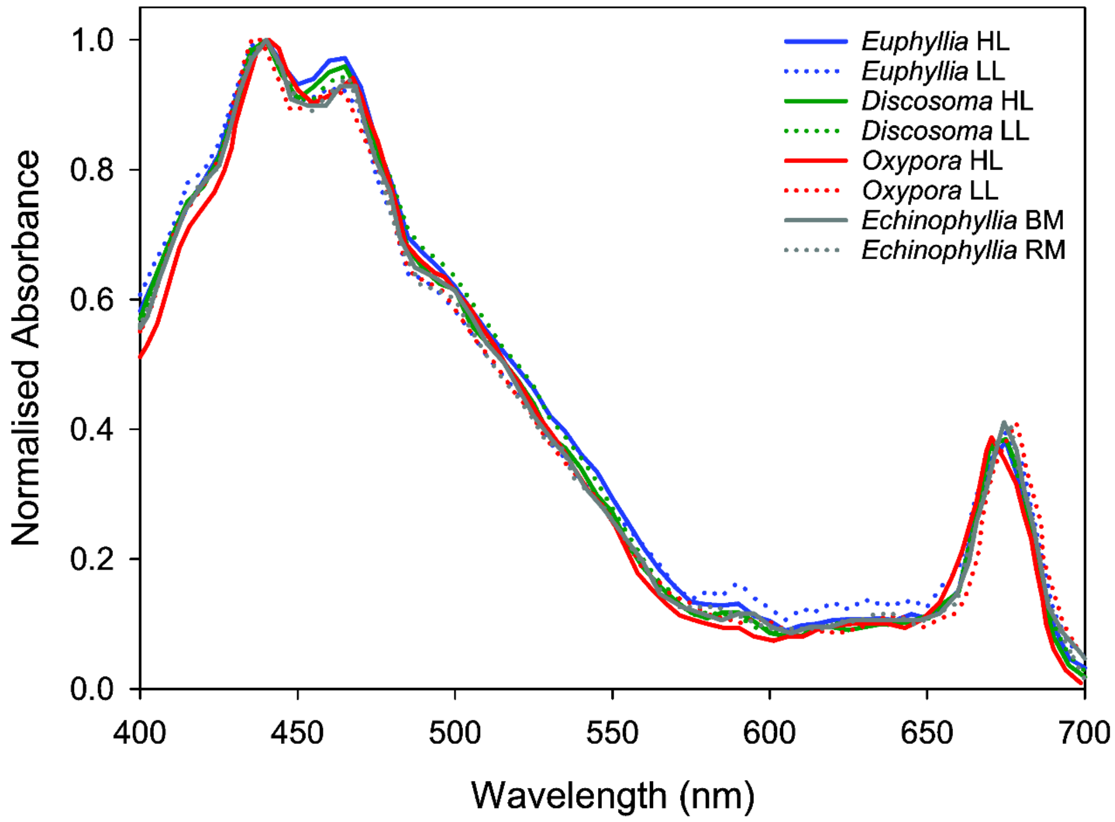

Supplementary Figure 4. Absorption spectra of zooxanthellae pigments in aqueous solution from different hosts and under different light conditions. HL, High Light; LL, Low Light; BM, Brown Morph; RM, Red Morph.

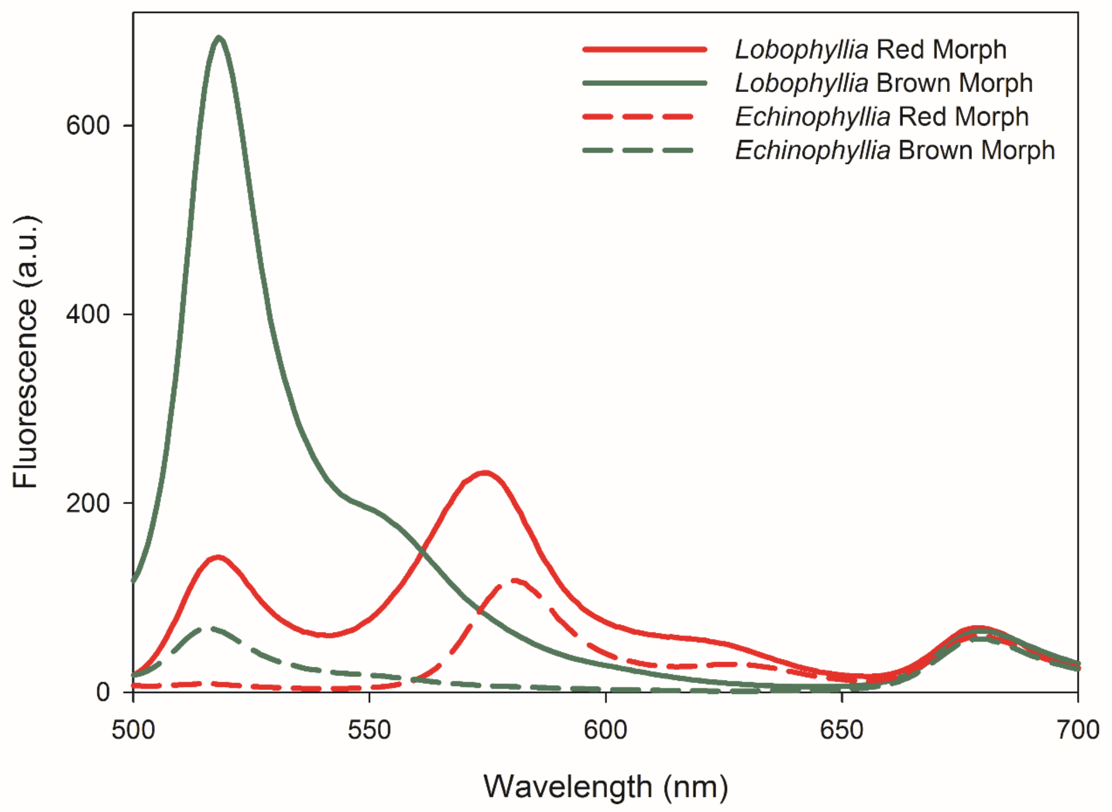

Supplementary Figure 5. Red fluorescence of colour morphs of *Lobophyllia hemprichii* and *Echinophyllia* sp. used in the light manipulation experiment. Spectra represent means of five independent measurements.

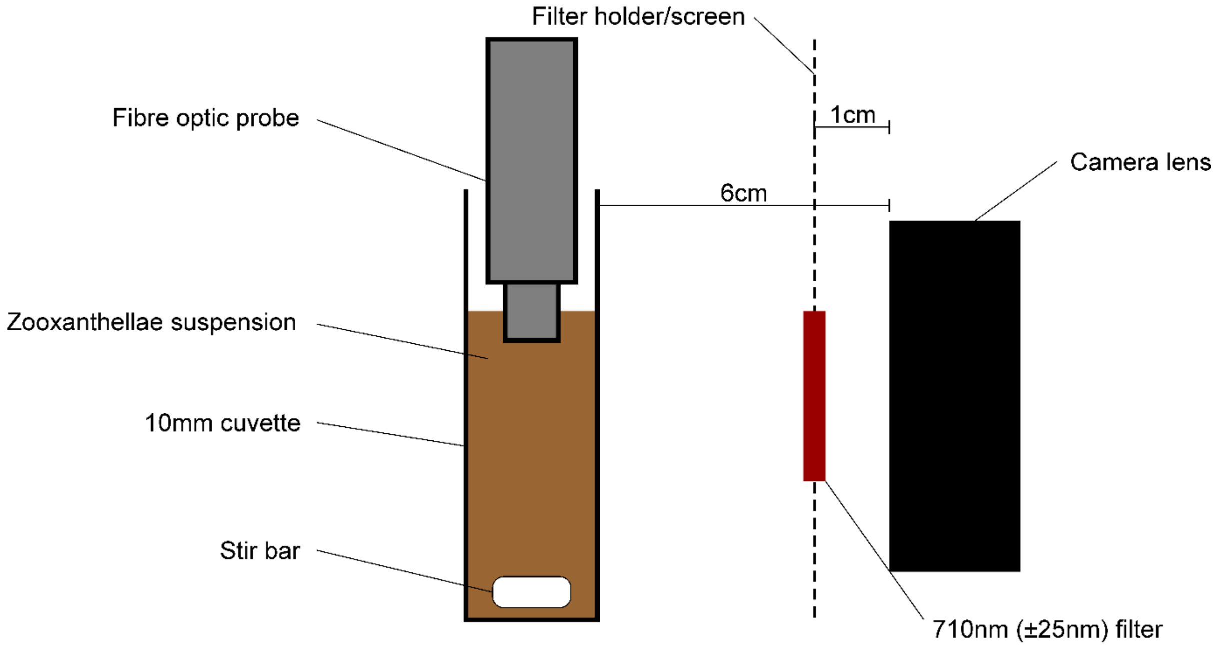

Supplementary Figure 6. A schematic diagram of the experimental setup for chlorophyll fluorescence imaging. The zooxanthellae suspension was illuminated with 20nm wide spectral bands, centred at wavelengths from 400nm to 660nm using the fibre optic probe. Images of the resulting chlorophyll fluorescence were collected through a narrow band pass filter (710 ±25nm). The diagram is not drawn to scale and specified distances are approximate. The setup was maintained across all wavelengths, as the images were collected in a consecutive manner; only the wavelength of illumination and the exposure times were modified between images.

Supplementary Table 1. Table of camera settings used in the acquisition of chlorophyll fluorescence images from *Discosoma sp.* and the zooxanthellae suspension. The f-stop provided is the lens setting used for the images but the effective f-stop will be greater (consistent for all images) due to the increase of the focal length associated with the macro extension tubes used.

	Discosoma sp.			**Zooxanthellae suspension**		
**Wavelength (nm)**	**F-Stop**	**Exposure Time (secs)**	**ISO**	**F-Stop**	**Exposure Time (secs)**	**ISO**
400	f/1.8	420	100	f/1.8	480	200
420	f/1.8	300	100	f/1.8	240	200
440	f/1.8	300	100	f/1.8	240	200
460	f/1.8	300	100	f/1.8	180	200
480	f/1.8	300	100	f/1.8	180	200
500	f/1.8	420	100	f/1.8	240	200
520	f/1.8	420	100	f/1.8	300	200
540	f/1.8	420	100	f/1.8	300	200
560	f/1.8	420	100	f/1.8	420	200
580	f/1.8	660	100	f/1.8	600	200
600	f/1.8	660	100	f/1.8	720	200
620	f/1.8	780	100	f/1.8	720	200
640	f/1.8	780	100	f/1.8	720	200
660	f/1.8	780	100	f/1.8	720	200

Supplementary Table 2. Mean number of colonies sampled at different depths in the Gulf of Eilat.

**Depth (m)**	**Mean number of colonies sampled (S.D.)**
1	301 *(±84)*
5	46 *(±24)*
12	42 *(±15)*
20	53 *(±28)*
30	203 *(±35)*
45	100 *(±16)*

References

1. Rosset S., Wiedenmann J., Reed A.J., D'Angelo C. 2017 Phosphate deficiency promotes coral bleaching and is reflected by the ultrastructure of symbiotic dinoflagellates. *Marine pollution bulletin* **118**(1), 180-187.

2. Sharon Y., Levitan O., Spungin D., Berman-Frank I. 2011 Photoacclimation of the seagrass Halophila stipulacea to the dim irradiance at its 48-meter depth limit. *Limnology and Oceanography* **56**(1), 357.
